# The Soybean *Rfg1* Gene Restricts Nodulation by *Sinorhizobium fredii* USDA193

**DOI:** 10.3389/fpls.2017.01548

**Published:** 2017-09-07

**Authors:** Yinglun Fan, Jinge Liu, Shanhua Lyu, Qi Wang, Shengming Yang, Hongyan Zhu

**Affiliations:** ^1^College of Agriculture, Liaocheng University Liaocheng, China; ^2^Department of Plant and Soil Sciences, University of Kentucky, Lexington KY, United States

**Keywords:** soybean, nodulation, nitrogen fixation, rhizobial symbiosis, symbiosis specificity

## Abstract

*Sinorhizobium fredii* is a fast-growing rhizobial species that can establish a nitrogen-fixing symbiosis with a wide range of legume species including soybeans (*Glycine max*). In soybeans, this interaction shows a high level of specificity such that particular *S. fredii* strains nodulate only a limited set of plant genotypes. Here we report the identification of a dominant gene in soybeans that restricts nodulation with *S. fredii* USDA193. Genetic mapping in an F2 population revealed co-segregation of the underlying locus with the previously cloned *Rfg1* gene. The *Rfg1* allele encodes a member of the Toll-interleukin receptor/nucleotide-binding site/leucine-rich repeat class of plant resistance proteins that restricts nodulation by *S. fredii* strains USDA257 and USDA205, and an allelic variant of this gene also restricts nodulation by *Bradyrhizobium japonicum* USDA122. By means of complementation tests and CRISPR/Cas9-mediated gene knockouts, we demonstrate that the *Rfg1* allele also is responsible for resistance to nodulation by *S. fredii* USDA193. Therefore, the *Rfg1* allele likely provides broad-spectrum resistance to nodulation by many *S. fredii* and *B. japonicum* strains in soybeans.

## Introduction

The leguminous plants are able to establish a symbiotic relationship with nitrogen-fixing soil bacteria called rhizobia. The symbiosis is featured by the formation of root nodules where the bacteria in nodule cells can convert atmospheric nitrogen into ammonia and make it available to the plant. This symbiotic partnership has important implications in sustainable agriculture because it reduces the need for nitrogen-based fertilizers.

The legume-rhizobial symbiosis starts with host perception of bacterially derived lipo-chitooligosaccharides known as nodulation (Nod) factors (Oldroyd et al., 2011). Recognition of Nod factors secreted by compatible bacteria induces cell divisions in the root cortex leading to the formation of nodule primordia, and at the same time initiates the infection process that delivers the bacteria into these primordia. Infection of most legumes such as soybeans (*Glycine max*) and alfalfa (*Medicago sativa*) is through root hairs. Bacteria are first entrapped by curled root hairs and multiply to form micro-colonies referred to as infection foci. From these foci, plant-made tubular-like structures, called infection threads, start to develop through local cell wall hydrolysis and invagination of the plant plasma membrane. The infection threads that are colonized by dividing bacteria proceed through the epidermal cell layer into the inner cortex where the nodule primordium has formed. Bacteria are then internalized in an endocytosis-like process and surrounded by a host membrane, where they differentiate into nitrogen-fixing bacteroides and are confined in organelle-like structures called symbiosomes (Jones et al., 2007; Oldroyd et al., 2011).

While some bacteria can nodulate a wide range of hosts, most bacteria have strict host selectivity. As such, particular rhizobial species or strains nodulate only a narrow group of legume species or genotypes (Broughton et al., 2000; Perret et al., 2000; Wang et al., 2012). Understanding the genetic and molecular basis of this specificity is important for developing strategies to improve the agronomic potential of the root nodule symbiosis in agriculture. It has been reported that plant domestication and breeding processes have led to the reduced ability of the modern cultivars to interact with indigenous soil strains as compared to their wild progenitors (Mutch and Young, 2004; Kiers et al., 2007; Kim et al., 2014). In this case, development of cultivars that are promiscuous with indigenous strains would be beneficial. On the other hand, certain indigenous soil bacteria are highly competitive for nodulation with host legumes but with low nitrogen fixation efficiency. Under this latter scenario, it is desirable to grow plants that can restrict nodulation with low-efficient indigenous strains but nodulate preferentially with the effective inoculant strains (Keyser and Li, 1992; Devine and Kuykendall, 1996).

Establishment of a root nodule symbiosis requires mutual recognition of multiple molecular signals between the symbiotic partners (Jones et al., 2007; Deakin and Broughton, 2009; Oldroyd et al., 2011). Therefore, symbiotic specificity can be regulated by multiple mechanisms at different stages of the nodule development (Perret et al., 2000; Wang et al., 2012, 2017; Liu et al., 2014; Yang et al., 2017). In most legumes, bacterial infection and nodule formation is initiated by host recognition of rhizobial Nod factors (Lerouge et al., 1990; Geurts et al., 1997; Limpens et al., 2003; Radutoiu et al., 2003). Nod factors produced by different bacteria carry specific chemical decorations on the chitin backbone, and this structural diversity has been thought to be a major determinant of nodulation specificity in the legume–rhizobal interaction, particularly at the species level (Bras et al., 2000; Radutoiu et al., 2007). Similar to pathogenic bacteria, symbiotic rhizobia also use conserved microbe-associated molecular patterns (MAMPs) or secreted effectors to facilitate their interaction with the host (D’Haeze and Holsters, 2004; Fauvart and Michiels, 2008; Deakin and Broughton, 2009; Soto et al., 2009; Downie, 2010; Wang et al., 2012; Kawaharada et al., 2015). Accordingly, effector- or MAMP-triggered plant immunity mediated by host receptors also plays an important role in regulating host range of rhizobia (Yang et al., 2010; Wang et al., 2012; Faruque et al., 2015; Kawaharada et al., 2015; Tang et al., 2016).

We have cloned several dominant genes in soybeans (e.g., *Rj2*, *Rfg1*, and *Rj4*) that restrict nodulation with specific rhizobial strains (Yang et al., 2010; Tang et al., 2016). In these cases, symbiosis incompatibility is controlled in a similar manner as ‘gene-for-gene’ resistance against plant pathogens (Sadowsky et al., 1990; Devine and Kuykendall, 1996). *Rj2* and *Rfg1* are allelic genes, each encoding a typical Toll-interleukin receptor/nucleotide-binding site/leucine-rich repeat (TIR-NBS-LRR) resistance protein that confers resistance to nodulation by specific strains of *Bradyrhizobium japonicum* and *Sinorhizobium fredii*, respectively (Yang et al., 2010), while *Rj4* encodes a thaumatin-like pathogenesis-related protein that restricts nodulation by specific strains of *B. elkanii* (Tang et al., 2016). Moreover, the function of these nodulation-restrictive genes is dependent on the bacterial type III secretion system (Krishnan et al., 2003; Okazaki et al., 2009; Yang et al., 2010; Tsukui et al., 2013; Tsurumaru et al., 2015; Tang et al., 2016; Yasuda et al., 2016). These studies revealed an important role of effector-triggered plant immunity in the regulation of nodulation specificity in soybeans (Wang et al., 2012).

Here we describe the study of symbiotic incompatibility of soybean with *S. fredii* USDA193. A previous report suggested that restriction of nodulation by this strain is associated with the *Rj2* and/or *Rj3* loci (Nakano et al., 1997). Our study revealed a single dominant gene responsible for this incompatibility. Genetic mapping in an F2 population showed co-segregation of the underlying locus with the previously cloned *Rfg1* gene that confers resistance to nodulation by *S. fredii* strains USDA257 and USDA205 (Yang et al., 2010). Through complementation tests and CRISPR/Cas9-mediated gene disruption, we demonstrate that the *Rfg1* allele also is responsible for restricting nodulation by *S. fredii* USDA193. Our study suggests that the *Rfg1* locus is involved in the determination of nodulation specificity with multiple *S. fredii* and *B. japonicum* strains in soybeans.

## Materials and Methods

### Plant Material, Nodulation Assay and Genotyping

The F2 mapping population was derived by crossing the North American cultivar ‘Williams 82’ with the cultivar ‘Peking,’ an introduction from China. Peking formed nitrogen-fixing nodules when inoculated with *S. fredii* USDA193 (Nod+) while Williams 82 restricted nodulation by this strain (Nod-) (Keyser et al., 1982). *S. fredii* USDA193, originally isolated from China (Keyser et al., 1982), was obtained from the National *Rhizobium* Germplasm Collection (USDA-ARS, Beltsville, MD, United States). The strain was cultured on YEM agar plates (yeast extract, 1.0 g/L; mannitol, 10.0 g/L; dipotassium phosphate, 0.5 g/L; magnesium sulfate, 0.2 g/L; sodium chloride, 0.1 g/L; calcium carbonate, 1.0 g/L; agar, 15.0 g/L) in the dark at 28°C for 4–5 days, and the bacterial paste was then collected and diluted in sterile water to OD_600_ of 0.1. For nodulation assay, each 1-week-old seedling was flood-inoculated with 10 mL of the bacterial suspension. Plants were grown in sterilized 50/50 Perlite-Turface mix in a growth chamber programmed for 16 h light at 26°C and 8 h dark at 23°C. Nodulation phenotype was assayed 4 weeks post inoculation. Genotyping was conducted by using a CAPS (cleaved amplified polymorphic sequences) marker developed based on a SNP (single nucleotide polymorphism) between the *Rfg1* (Williams 82) and *rfg1* (Peking) alleles. The primer pair used was 5′TGAGAGTACTGGAATGGTGGAG3′ and 5′TTGCTGATCGAACCACTCTG3′, and the restriction enzyme used was *Hpa*I.

### Complementation Tests

The Williams 82 allele of *Rfg1* was used for complementation tests. Genomic DNA of the *Rfg1* allele was derived from the BAC clone Gm_WBa0019D20 of Williams 82 (Marek and Shoemaker, 1996) by digestion with *Pst*I and *Bmg*BI. The released 10.9-kb fragment included the 4.9-kb coding region, the 4.0 kb upstream of the start codon containing the promoter and 5′ untranslated region, and the 2.0 kb downstream of the stop codon encompassing the 3′ untranslated region. The DNA fragment was cloned into the binary vector pCAMBIA1305.1 through blunt end cloning. The binary vector was transferred into the Peking genetic background through hairy root transformation as described below. The transgenic roots were identified by GUS-staining because of the presence of a GUS expression cassette in the pCAMBIA1305.1 vector.

### CRISPR/Cas9-Mediated Gene Knockout

The CRISPR/Cas9 gene knockout constructs were developed based on the pHSE401 vector described by Xing et al. (2014). Two pairs of oligos were designed to specifically target two different sites within the fourth exon of *Rfg1*. For the first targeted site, we used the oligo pair 5′ATTGATGAGGACTTAAAAAGCTC3′ and 5′AAACGAGCTTTTTAAGTCCTCAT3′. For the second targeted site, we used the oligo pair 5′ATTGACAGTAAGC CTTACTACCT3′ and 5′AAACAGGTAGTAAGGCTTACTGT3′. The underlined sequences represent the targeted positions. The oligo pairs were first annealed to produce a double-stranded fragment with 4-nt 5′ overhangs at both ends, and then ligated into the *Bsa*I-digested pHSE401 vector. The constructs were individually transformed to the Williams 82 (Nod-) background by means of hairy root transformation. To validate the CRISPR/Cas9-mediated gene disruption, the roots that formed nodules were subjected to DNA isolation, PCR amplification, and DNA sequencing. If the initial sequencing suggested the presence of multiple heterogeneous mutant alleles, the PCR product was ligated into pGEM T-Easy Vector System (Promega) and at least 10 colonies were selected for sequencing.

### Construction of a Chimeric Gene of *Rfg1* and *Rj2*

The *Rfg1* allele that restricts nodulation by *S. fredii* USDA257 is allelic to *Rj2*, an allele that restricts nodulation by *B. japonicum* USDA122 (Yang et al., 2010). At this locus, there exist three type of alleles in natural populations of soybeans, including the *Rj2 (rfg1*) allele that restricts nodulation with USDA122 but allows nodulation with USDA257, the *rj2* (*Rfg1*) allele that permits nodulation with USDA122 but restricts nodulation with USDA257, and the *rj2* (*rfg1*) allele that nodulates with both strains. These allelic specificities are defined by seven amino-acid substitutions (Yang et al., 2010). However, we did not identify an *Rj2* (*Rfg1*) allele type that prohibits nodulation with both USDA122 and USDA257 in the surveyed soybean lines (Yang et al., 2010). We thus modified the *Rfg1* allele by replacing part of polymorphic sequence of *Rfg1* with the corresponding sequence of the *Rj2* allele, with the expectation to generate a chimeric gene that could lead to restriction of nodulation by both strains. For this purpose, we amplified two overlapping DNA fragments, one that contained the substitutions responsible for the *Rj2* allelic function and another that possessed the substitutions required for the *Rfg1* allelic function. We then used an overlapping PCR strategy to assemble the two DNA templates into a single one. The amplified fragment was then cloned into the *EcoR*I digested genomic construct of *Rfg1* mentioned above using the In-Fusion Advantage PCR Cloning Kits (Clontech). We tested the function of this chimeric allele by transferring it into Peking, a genotype that formed nodules with both USDA122 and USDA257. To avoid redundancy, we will provide a further description of this experiment in the “Results” section.

### Hairy Root Transformation

*Agrobacterium rhizogenes*-mediated hairy root transformation was carried out based on the protocol described by Kereszt et al. (2007). Briefly, bacterial paste of the *A. rhizogenes* strain K599 that contains individual binary vectors was injected into the cotyledonary node of 1-week-old seedlings using a latex free syringe with a thin needle (0.4 mm × 13 mm) (1 ml 27G1/2, Becton, Dickinson & Co.). The infected seedlings were grown in sterile vermiculite and covered with plastic bags in a growth chamber to maintain high humidity. Two to three weeks after inoculation, when hairy roots were well developed at the infection sites, the main roots were removed, and the composite plants were inoculated with rhizobia. Nodulation assays were performed 4 weeks post inoculation.

### Microscopic Analysis

Assay for root hair curling followed the method described in Yang et al. (2010). For anatomical analysis of nodules, nitrogen-fixing nodules of Peking and rudimentary nodules of Williams 82 were harvested 4 weeks post inoculation and immediately fixed in 4% paraformaldehyde (w/v) overnight at 4°C. The tissues were then dehydrated in a graded ethanol series followed by a graded series of xylene. After infiltrated in 50/50 Epon-Araldite resin and propylene oxide overnight and then in 75/25 Epon-Araldite resin and propylene oxide for 8 h, the samples were embedded in resin. Embedded tissues were sectioned (10 μm thick) with a microtome, stained with Toluidine Blue, and examined with bright-field optics.

## Results

### Characterization of the Soybean–*S. fredii* USDA193 Interactions

**FIGURE 1:**
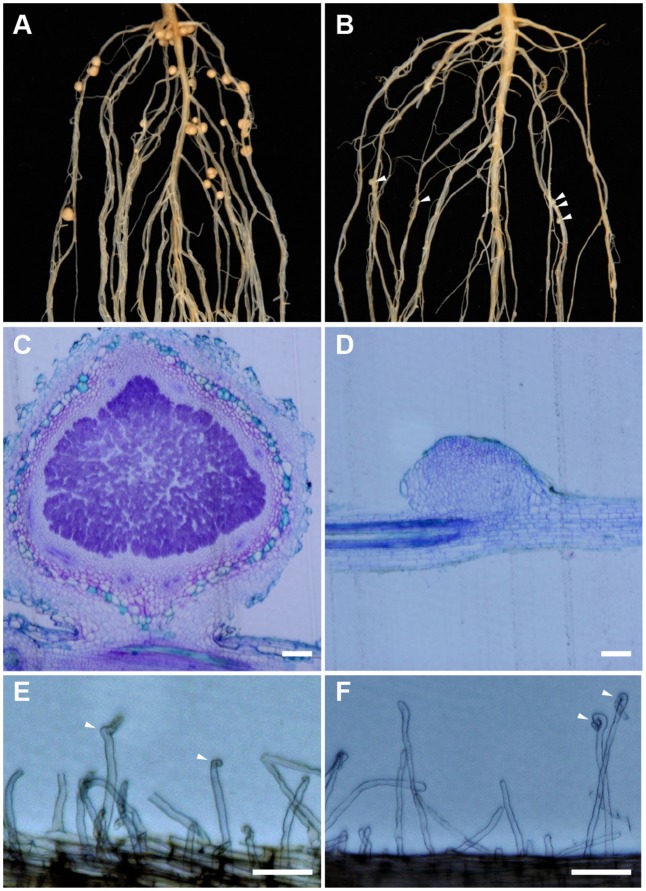
Nodulation specificity associated with *Sinorhizobium fredii* USDA193 in soybean. The bacterial strain formed nitrogen-fixing nodules on Peking **(A)** but only small nodule primordia on Williams 82, as indicated by the arrowheads **(B)**. In the compatible Peking/USDA193 interaction, nodules developed normally and contained bacteria **(C)**, whereas in the incompatible interaction between Williams 82 and USDA193, the nodule primordia did not contain bacteria **(D)**. Bars = 100 μm. However, USDA193 induced root hair curling (indicated by arrowheads) on both Peking **(E)** and Williams 82 **(F)**. Photographs were taken 5 days post inoculation. Bars = 100 μm.

*Sinorhizobium fredii* USDA193 formed mature nitrogen-fixing nodules on the roots of Peking (**Figure 1A**) but not on the roots of Williams 82 (**Figure 1B**). Despite being unable to form functional nodules in the Williams 82 background, the bacterial strain could frequently induce the formation of rudimentary nodules, bump-like small cortical proliferations on the roots (**Figure 1B**). In contrast to the infected nodules formed in the compatible interaction (**Figure 1C**), the rudimentary nodules on the roots of Williams 82 were completely devoid of infected bacteria; as such, the cortical cell division ceased at very early stage of the nodule development (**Figure 1D**). The ability to produce rudimentary nodules with USDA193 on the Williams 82 roots suggested that the early responses of Nod factor perception are not affected. Consistent with this inference, root hair curling, a hallmark of Nod factor responses, occurred in both compatible and incompatible interactions (**Figures 1E,F**). Thus, we concluded that the restriction of nodulation by *S. fredii* USDA193 in Williams 82 was not due to a failure in Nod factor perception but caused by the block of bacterial infection.

### Restriction of Nodulation with USDA193 Is Controlled by a Single Dominant Gene Mapped to the *Rfg1* Locus

We carried out genetic analysis of the symbiosis incompatibility involving *S. fredii* USDA193 in an F2 population derived from the cross between Williams 82 (Nod-) and Peking (Nod+). From a small population of 122 plants inoculated by USDA193, 95 were Nod- and 27 were Nod+. The segregation statistically fits the 3:1 (Nod- to Nod+) ratio (χ^2^ = 0.54, *df* = 1, *P* = 0.46), suggesting that the restriction of nodulation by USDA193 in Williams 82 is controlled by a single dominant gene. The dominant nature of the nodulation-restrictive allele supports our hypothesis that the incompatible interaction between Williams 82 and USDA193 was not due to a failure in Nod factor signaling but resembles ‘gene-for-gene’ resistance in the plant–pathogen interactions.

Williams 82 and Peking showed the same phenotype when inoculated with *S. fredii* strains USDA257 and USDA193, and we previously reported that the resistance to nodulation with USDA257 in Williams 82 is controlled by the dominant *Rfg1* allele (*Glyma16g33780*) that encodes a TIR-NBS-LRR protein (Yang et al., 2010). We therefore suspected that *Rfg1* possibly also confers resistance to *S. fredii* USDA193. To test this possibility, we started the mapping experiment by using a polymorphic DNA marker developed from *Rfg1*. Consistent with our hypothesis, linkage analysis in the aforementioned F2 population revealed co-segregation between the nodulation phenotypes and the marker genotypes. Thus, we considered *Rfg1* as a candidate gene that restricts nodulation by *S. fredii* USDA193.

### *Rfg1* Is Responsible for Resistance to Nodulation by *S. fredii* USDA193

**FIGURE 2:**
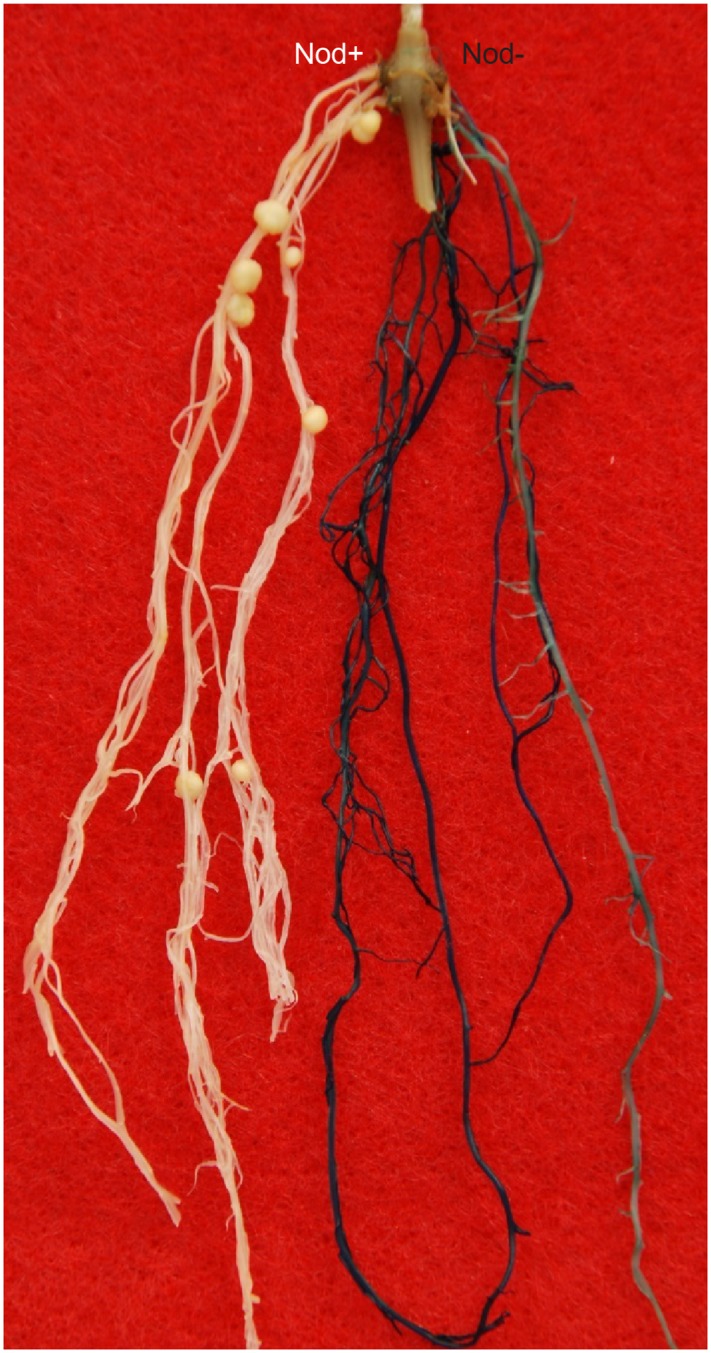
Introduction of the *Rfg1* allele of Williams 82 into Peking led to restriction of nodulation on the transgenic roots by USDA193 (blue), but nodulation was normal on the wild-type roots (white).

We first tested the *Rfg1* gene by complementation tests using *A. rhizogenes*-mediated hairy root transformation. Because the transformation experiments were performed without selection, the hairy roots induced by *A. rhizogenes* included both transgenic and wild type, which can be readily distinguished by the GUS-staining assay. As shown in **Figure 2**, introduction of the *Rfg1* allele of Williams 82 into the Peking background resulted in complete block of nodule formation on the transgenic roots. From >20 composite transgenic plants that possessed both transgenic and wild-type roots, nodules were formed on the wild-type roots but not on the transgenic roots.

**FIGURE 3:**
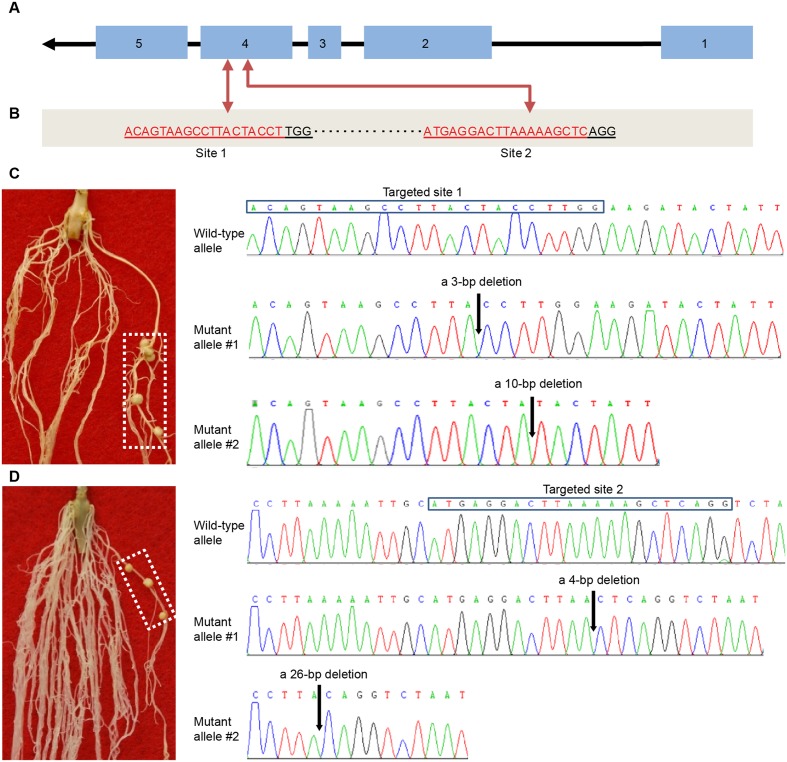
CRISPR/Cas9-mediated knockout of *Rfg1* in the Williams 82 (Nod–) background. **(A)** Gene structure of *Rfg1*. The exons and introns are indicated by boxes and lines, respectively. Arrow indicates the transcription direction. **(B)** Two targeted sites on the fourth exon. The protospacer adjacent motif (PAM) is ‘TGG’ for the site 1 and ‘AGG’ for the site 2. **(C)** An example showing that knockout of *Rfg1* at the site 1 led to the formation of root nodules on a transgenic root (boxed). Sequence analysis revealed that this root contained two mutant alleles, one with a 3-bp deletion and another with a 10-bp deletion (indicated by arrows). **(D)** An example showing that knockout of *Rfg1* at the site 2 also resulted in the formation of root nodules on a transgenic root (boxed). Sequence analysis of the DNA from this root identified two mutant alleles, one with a 4-bp deletion and another with a 26-bp deletion (indicated by arrows).

We further used the CRISPR/Cas9-based reverse genetics tool (Doudna and Charpentier, 2014) to knock out the *Rfg1* gene in the Williams 82 background (Nod-). For this purpose, we designed two gRNA vectors that individually target two different sites of the fourth exon (**Figures 3A,B**). The vectors were introduced to *A. rhizogenes* K599 for hairy root transformation, followed by assaying the nodulation capacity of the hairy roots by inoculation with *S. fredii* USDA193. For both vectors, we obtained >30 putative transgenic roots from more than 50 independent plants that formed mature nitrogen-fixing nodules. DNA sequencing validated the targeted gene disruption in these roots and these roots did not contain the wild-type allele. Two examples are illustrated in **Figures 3C,D**. **Figure 3C** shows that a transgenic root forming nodules resulted from the knockout of *Rfg1* at the site 1, and sequence analysis revealed two mutant alleles, one with a 3-bp deletion and another with a 10-bp deletion. **Figure 3D** represents an example showing that knockout of *Rfg1* at the site 2 also led to the formation of root nodules on a transgenic root; sequence analysis identified two mutant alleles, one with a 4-bp deletion and another with a 26-bp deletion when compared with the wild-type allele. Taken together, we conclude that *Rfg1* is responsible for nodulation restriction by *S. fredii* USDA193 in Williams 82.

### Functional Analysis of a Chimeric Gene of *Rfg1* and *Rj2*

**FIGURE 4:**
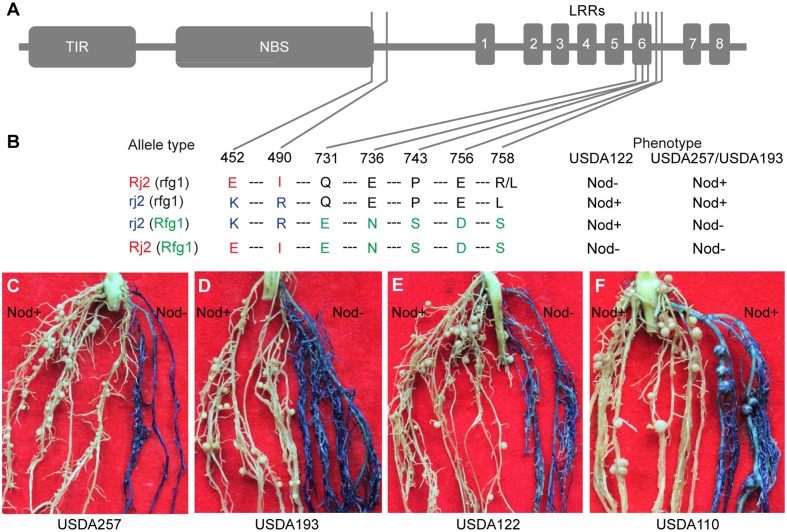
Functional analysis of a chimeric gene of *Rfg1* and *Rj2*. **(A,B)** Domain structure of the TIR-NBS-LRR protein **(A)** showing the seven substitution sites and amino acid polymorphisms that distinguish between the Rj2 (rfg1), rj2 (rfg1), and rj2 (Rfg1) protein isoforms **(B)**. Rj2 (Rfg1) represents a protein isoform resulting from expressing the chimeric gene. **(C–F)** Transgenic roots expressing the chimeric gene (blue) in the Peking background restricted nodulation by USDA257 **(C)**, USDA193 **(D)**, and USDA122 **(E)** but allowed nodulation with *B. japonicum* USDA110 **(F)**. In all cases, the nodulation was normal on the non-transgenic roots (white).

*Rfg1* and *Rj2* are allelic genes, each encoding a TIR-NBS-LRR protein of 1052 amino acids (Yang et al., 2010). A survey of a group of soybean lines identified three types of naturally occurring alleles, namely *Rj2 (rfg1)*, *rj2* (*Rfg1*), and *rj2* (*rfg1*) (Yang et al., 2010). The *Rj2 (rfg1*) allele restricts nodulation with USDA122 but not with USDA257; the *rj2* (*Rfg1*) allele restricts nodulation with USDA257 but not with USDA122; and the *rj2* (*rfg1*) allele allows nodulation with both strains. These allelic specificities are determined by seven amino acid substitutions occurring around the C-terminus of the NBS domain and the sixth LRR repeat (**Figures 4A,B**). Comparing between Rj2 (rfg1) and rj2 (rfg1) and between rj2 (Rfg1), and rj2 (rfg1) suggests that E452 and I490 are required for the *Rj2*-mediated nodulation restriction against USDA122, while E731, N736, S743, D756, and S758 are essential for *Rfg1*-mediated resistance against USDA257 and USDA193. If this inference is true, then the chimeric gene of *Rj2* and *Rfg1*, called *Rj2* (*Rfg1*), encoding a protein with E452, I490, E731, N736, S743, D756, and S758, would prevent nodulation with both strains. We generated multiple transgenic roots (from >100 independent plants) expressing the chimeric gene in Peking that carries an *rj2* (*rfg1*) allele and forms nodules with USDA257, USDA193, and USDA122. In consistence with our hypothesis, the transgenic roots restrict nodulation with both USDA257 (**Figure 4C**), USDA193 (**Figure 4D**), and USDA122 (**Figure 4E**). The transgenic roots retained their ability to nodulate with *B. japonicum* USDA110, a strain that nodulates both *Rj2* and *Rfg1* genotypes (**Figure 4F**).

## Discussion

Previous studies showed that the soybean *Rfg1* gene restricts nodulation with the fast-growing *S. fredii* strains USDA257 and USDA205 (Devine and Kuykendall, 1996; Yang et al., 2010). The *Rfg1*-mediated resistance to USDA257 was dependent on the bacterial type III secretion system and presumably resulted from the effector-triggered plant immunity, even though the cognate effector(s) has not yet been identified (Krishnan et al., 2003; Yang et al., 2010). In this paper, we demonstrate that the soybean *Rfg1* allele also conditions nodulation restriction with *S. fredii* USDA193, suggesting that *Rfg1* likely provide broad-spectrum resistance against a proportion of *S. fredii* strains (Keyser et al., 1982).

The soybean *Rj2/Rfg1* locus confers nodulation specificity toward many naturally occurring *B. japonicum* and *B. fredii* strains (Caldwell, 1966; Devine and Kuykendall, 1994; Trese, 1995; Yang et al., 2010). A survey of 847 soybean genotypes from Asian countries revealed an *Rj2* allele frequency of 0.02, mainly originated from southeast China (Devine and Breithaupt, 1981). In contrast, the *Rfg1* allele occurred with a much higher frequency of 0.44 in a similar population consisting of 285 plant introductions from Asian countries (Devine, 1985). The distribution of the *Rfg1* (0.44) and *rfg1* (0.56) allele frequencies suggests that these alleles has not been subjected to strong natural selection. However, a screen of 197 soybean lines from the Midwestern United States revealed an *Rfg1* allele frequency of 0.83, suggesting that the compatibility with *S. fredii* strains may be eroded during the breeding process, likely due to the narrow genetic basis of germplasms used in the soybean breeding programs (Balatti and Pueppke, 1992). The diversification of the *Rfg1* and *rfg1* alleles appears to predate that of the *Rj2* and *rj2* alleles. The *Rj2* (*rfg1*) allele likely have derived from the *rj2 (rfg1)* allele through gain-of-function mutations. The *rj2 (rfg1)* allelic form is most promiscuous, conferring an unrestricted nodulation phenotype with many strains that are restricted by the *Rj2* or *Rfg1* alleles. Therefore, the *rj2 (rfg1)* genotype should be prioritized in soybean breeding programs if the goal is to ensure the cultivar to be able to nodulate with indigenous bacterial strains.

The polymorphic *Rj2*/*Rfg1* locus encodes a TIR-NB-LRR gene with three allelic variants that show differing specificities to yet unknown bacterial effectors. E452 and I490 in or near the C-terminal NBS domain of Rj2 are required for the recognition of the cognate effector from *B. japonicum* USDA122, while E731, N736, S743, D756, and S758 in the LRR domain of Rfg1 are essential for the recognition of the corresponding effectors secreted by USDA257 and USDA193. This situation is similar to the previous studies showing that both TIR, NBS, and LRR domains could be involved in the determination of resistance specificity (Ellis et al., 1999; Dodds et al., 2001; Mondragón-Palomino et al., 2002; Ellis et al., 2007). The chimeric *Rj2(Rfg1)* allele composed of E452, I490, E731, N736, S743, D756, and S758 could recognize corresponding effectors of all these strains. Such an allele could exist in natural population if intragenic recombination events occur between the *Rj2(rfg1)* and *rj2(Rfg1)* alleles. It is possible that these polymorphic sites play a role in direct effector binding or intra- or intermolecular interactions in protein complexes during recognition and signaling. Further analysis of the mechanisms underlying the recognitions mediated by the *Rj2/Rfg1* locus awaits the identification of the cognate bacterial effectors.

## Author Contributions

YF, JL, SL, and HZ: conception and design of the work. YF, JL, QW, SY, and SL: performed the work and analyzed data. HZ wrote the paper.

## Conflict of Interest Statement

The authors declare that the research was conducted in the absence of any commercial or financial relationships that could be construed as a potential conflict of interest. The reviewer DR and handling Editor declared their shared affiliation, and the handling Editor states that the process nevertheless met the standards of a fair and objective review.
